# Differential pulse voltammetry and chronoamperometry as analytical tools for epinephrine detection using a tyrosinase-based electrochemical biosensor

**DOI:** 10.1039/d2ra04045j

**Published:** 2022-09-06

**Authors:** Sylwia Baluta, Francesca Meloni, Kinga Halicka, Adam Szyszka, Antonio Zucca, Maria Itria Pilo, Joanna Cabaj

**Affiliations:** Faculty of Chemistry, Wrocław University of Science and Technology Wybrzeże Wyspiańskiego 27 50-370 Wrocław Poland sylwia.baluta@pwr.edu.pl; Department of Chemistry and Pharmacy, University of Sassari Via Vienna 2 07100 Sassari Italy; Faculty of Microsystem Electronics and Photonics, Wrocław University of Science and Technology Wybrzeże Wyspiańskiego 27 50-370 Wrocław Poland

## Abstract

The main goal of the presented study was to design a biosensor-based system for epinephrine (EP) detection using a poly-thiophene derivative and tyrosinase as a biorecognition element. We compared two different electroanalytical techniques to select the most prominent technique for analyzing the neurotransmitter. The prepared biosensor system exhibited good parameters; the differential pulse (DPV) technique presented a wide linear range (1–20 μM and 30–200 μM), with a low detection limit (0.18 nM and 1.03 nM). In the case of chronoamperometry (CA), a high signal-to-noise ratio and lower reproducibility were observed, causing a less broad linear range (10–200 μM) and a higher detection limit (125 nM). Therefore, the DPV technique was used for the calculation of sensitivity (0.0011 μA mM^−1^ cm^−2^), stability (49 days), and total surface coverage (4.18 × 10^−12^ mol cm^−2^). The biosensor also showed very high selectivity in the presence of common interfering species (*i.e.* ascorbic acid, uric acid, norepinephrine, dopamine) and was successfully applied for EP determination in a pharmaceutical sample.

## Abstract 2

GCE/poly-4,4′-bBT/tyrosinase biosensor for epinephrine was constructed. Comparison of differential pulse voltammetry (DPV) and chronoamperometry was performed. DPV showed more reproducible results giving high selectivity, sensitivity, stability.

## Graphical abstract

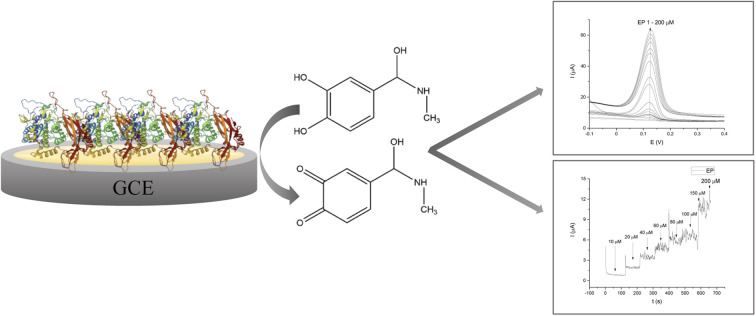

## 1. Introduction

Epinephrine (EP), a catecholamine that acts as a neurotransmitter in the nervous system, plays an important role as a chemical messenger between cells.^1^ Any dysfunction of the neurotransmitter level in nervous/metabolic processes can lead to a number of serious diseases, mainly neurodegenerative.^2–4^ Constant and fast monitoring of neurotransmitters would be very helpful in modern medical diagnostics, where biosensors could be used as potential point-of-care testing devices, as an alternative to classical analytical techniques for the detection of important analytes. Since 1962, when the first biosensor for glucose detection was constructed, intensive research has been carried out in the biosensor area.^5^ Biosensors help to assess the levels of biological markers or any chemical reaction by producing signals that are mainly related to the concentration of an analyte in the chemical reaction. Predominantly applied and most conventional are electrochemical biosensors, which are based on transducing the biochemical events to electrical signals.^6–9^ Such devices are constructed *via* chemical and biological modification of an electrode, which is used as a solid support for the anchoring of biomolecules and electron transfer. Although biosensors use a diversity of biorecognition elements (*e.g.* DNA, antibodies), electrochemical determination techniques employ mainly enzymes, like oxidoreductases, due to their binding capabilities and biocatalytic activity.^10,11^ Oxidoreductases, such as laccase or tyrosinase, catalyze the redox reaction of a wide range of compounds which can be observed as, for instance, current changes.

Electrochemical biosensors utilize a variety of detection techniques, such as amperometry, where a reaction carried out during the investigation generates a measurable current signal.^12^ One example of amperometric measurements is voltammetry, which controls current changes resulting from the redox reactions of an electroactive species at the electrode at a fixed potential. The most common techniques used in such electrochemical sensors are cyclic voltammetry (CV), differential pulse voltammetry (DPV), and chronoamperometry (CA). CV allows obtaining information on the redox potential and electrochemical reaction rates, as well as enables the adsorption of compounds on the electrode surface (*e.g.* in the case of electropolymerization).^13^ In this method, the current is measured between the working electrode and the counter electrode and the voltage between the reference electrode and the working electrode.^13,14^ In DPV the potential perturbation, which consists of small pulses, is superimposed upon a staircase waveform. Accordingly, it shows a higher sensitivity in comparison with CV and selectivity due to the enhanced discrimination of faradaic currents.^15^ CA is based on the application of the square-wave potential to the working electrode and a steady state current is measured as a function of time, which may be used for time-dependent system characterization with high sensitivity.^13,14^

Inclusion of biologically active materials, like enzymes, into the electrode design results in the development of new amperometric approaches for biomolecules which can be non-electroactive (*e.g.* glutamate), or whose surface electrochemistry is too complicated to permit direct recognition, like glucose.^16^ Since a biosensor consists of two inherent elements, a biorecognition element and a transducer, the choice of a proper bioreceptor and electrode material is a key factor in a reasonable biosensor design.

Glassy carbon electrode (GCE) is a good candidate in electrochemical biosensor systems as a transducer element, because of electrochemical inertness, gas impermeability, electrical conductivity, and high chemical resistance.^17–19^ Since electrochemical measurement is based on electron transfer between the heterogeneous electrode-solution interface, the appropriate modification of the electrode surface results in an improvement of electrocatalytic properties, reproducibility of the system, sensitivity, and stability.^19–23^ Such treatments may also reduce the passivation of the electrode surface due to intermediates formed in the redox reaction.^24^ Organic semiconductors are interesting candidates for application at the interface between biological systems and electronics. Macrostructures can be tailored to interact with their aqueous biological surroundings while at the same time being able to interface with electronics. Conductive polymers are very often used to modify the transducer surface: enhanced electron transfer, compatibility with biological molecules, easy preparation, and high reproducibility make them a material that effectively increases the sensitivity and stability of sensor devices. S. Bonyadi *et al.* used a GC electrode modified with a polymeric graphitic-C_3_N_4_/polyaniline/CdO (mpg-C_3_N_4_/PANI/CdO) nanocomposite for epinephrine determination.^25^ Polyaniline (PANI) is widely used in sensing technology due to its π-conjugation system, which ensures a very large specific surface area.^26^ The sensor showed linear responses in the range of 0.05–80 μM and 100–1000 μM for EP with the limit of detection equal to 0.011 μM.^25^ Thiophenes represent very promising building blocks for polymers thanks to easy polymerization, conductivity, and high stability.^27^ In the work presented by C.-Y. Lai *et al.*, poly(3-hexylthiophene-*co*-3-thiopheneacetic acid) (P(3HT-*co*-3TAA)) was used for the modification of indium tin oxide (ITO) glass electrodes.^28^ Onto such matrix urease was immobilized for urea detection. Obtained biosensor showed linearity in the concentration range from 0.99 to 4.97 mM and could find application in real urea analysis, as the serum urea concentration is in the range of 1.3 to 3.5 mM.

In this study, we present a comparison of electrochemical techniques for epinephrine determination with a biologically active element. A glassy carbon electrode was modified with a poly-thiophene derivative (poly-4,4′-bis(2-methyl-3-butyn-2-ol)-2,2′-bithiophene, poly-4,4′-bBT), which acts as a matrix for tyrosinase immobilization and as an electron transfer mediator, allow for enzyme-dependent redox reaction observations and electrochemical characterization. To our knowledge, tyrosinase immobilization onto the poly-4,4′-bBT matrix has not been previously described. Presented method showed a highly sensitive, selective, quick, and simple analytical procedure.

## 2. Materials and methods

### 2.1. Reagents and materials

**Fig. 1:**
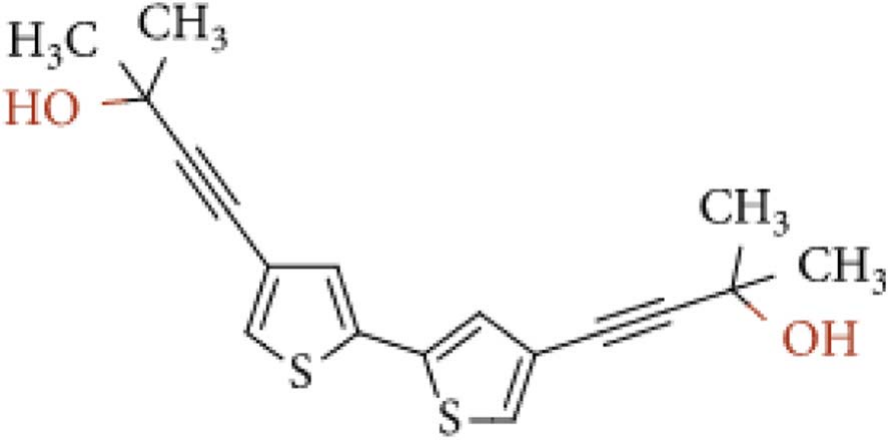
Chemical structure of 4,4′-bis(2-methyl-3-butyn-2-ol)-2,2′-bithiophene.

Tyrosinase (from *Agaricus bisporus*, EC 1.14.18.1, ≥1000 U mg^−1^), epinephrine hydrochloride (EP), tetrabutylammonium-tetrafluoroborate (TBA-TFB), dichloromethane, uric acid (UA), ascorbic acid (AA), and l-cysteine (CYS) were purchased from Sigma-Aldrich Co (Merck company). Citric acid (CA), NaOH, NaH_2_PO_4_, Na_2_HPO_4_, KH_2_PO_4_, Tris, HCl, CH_3_COONa, CH_3_COOH, NaCl, KCl, and glutaraldehyde (GA) were purchased from POCH (Part of Avantor, Performance Materials, Poland). The monomer – 4,4′-bis(2-methyl-3-butyn-2-ol)-2,2′-bithiophene (Fig. 1) was synthesized according to previous literature.^29^ All buffers were prepared according to generally known, obligatory standards. Drug *Adrenalinum* WZF 300 μg/0.3 mL was produced by a pharmaceutical company Polfa Warszawa, Poland.

### 2.2. Modification of electrode

**Fig. 2:**
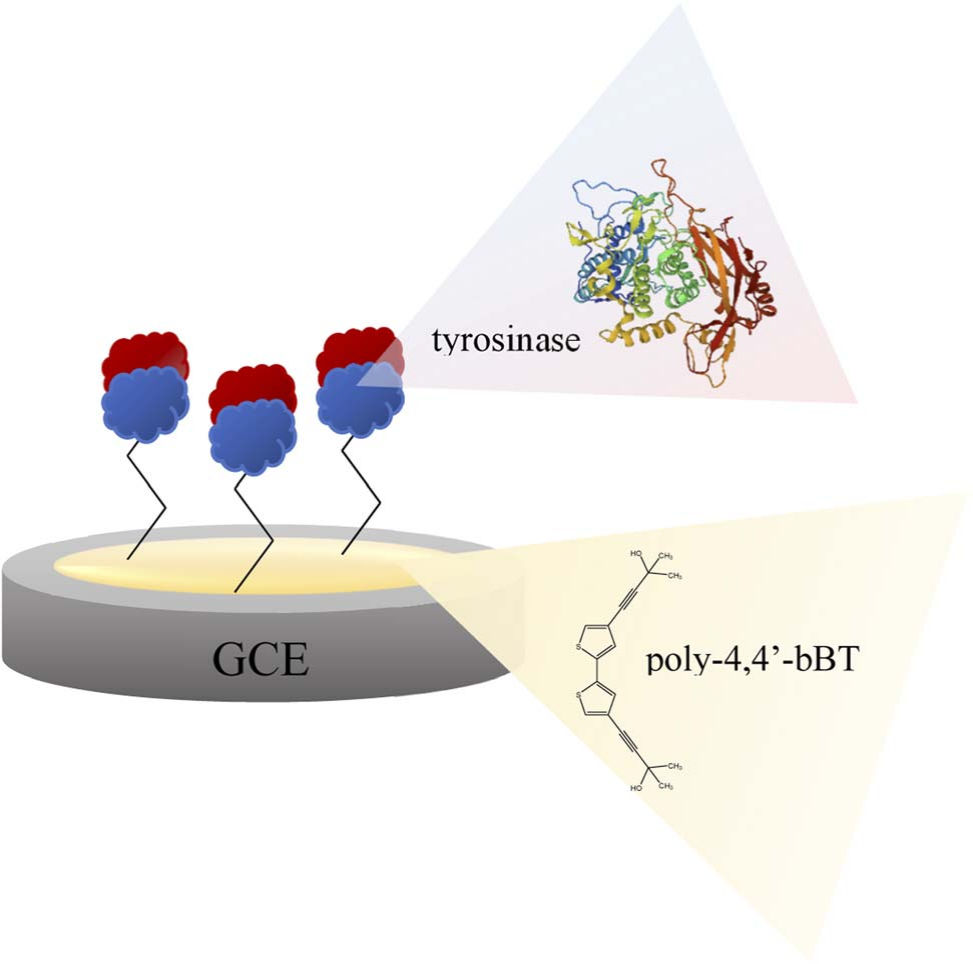
Scheme of the bioplatform for epinephrine determination – glassy carbon electrode modified with poly-4,4′-bBT and tyrosinase.

A glassy carbon electrode (GCE, diameter 3 mm, produced by BASi, MF-2012 model) was polished with a 50 nm polishing alumina suspension (BASi, CF-1050) and rinsed with double distilled water. Then, the electrode was electrochemically modified with a polymer layer of 4,4′-bis(2-methyl-3-butyn-2-ol)-2,2′-bithiophene (poly-4,4′-bBT) and tyrosinase (Fig. 2). The electrochemical synthesis of the polymer film was performed using a potentiostat/galvanostat AUTOLAB PGSTAT128N with NOVA software. A three-electrode electrochemical cell (10 mL) equipped with a glassy carbon as a working electrode (GCE), a silver–silver chloride as the reference electrode (Ag/AgCl), and a coiled platinum wire as the counter electrode, was used for all electrochemical experiments. Electrochemical synthesis of the polymeric layer onto a surface of the clean GCE was performed as follows: monomer (1 mM) was dissolved in a dichloromethane solution containing 0.1 M tetrabutylammonium-tetrafluoroborate (TBA-TFB) as a supporting electrolyte. The electrodes were dipped in 8 mL of the monomer solution. The polymer layers deposition was carried out through cyclic voltammetry (CV). The GC electrode was scanned in a potential range of 0.0–1.4 V *vs.* Ag/AgCl for 10 cycles, at a scan rate of 100 mV s^−1^. Then, the electrode modified with poly-4,4′-bBT was washed with dichloromethane. The stability measurement of obtained polymeric film was conducted by applying CV in the same conditions (potential range 0.0–1.4 V *vs.* Ag/AgCl, scan rate of 100 mV s^−1^) for 5 cycles in the supporting electrolyte solution.

In the next step, tyrosinase was immobilized on the modified electrode. Physical immobilization is a simple and inexpensive method, however, it poses a risk of potential desorption of the biocatalyst during measurements.^30^ To minimize this possibility, a cross-linker, *e.g.* glutaraldehyde (GA), is often employed for a more secure immobilization of enzymes onto the electrode surface. In this study, the immobilization process of tyrosinase onto the modified GCE was provided by physical adsorption with GA in PBS buffer (pH = 7.0) at room temperature. Physical adsorption lasted for 2 h, after which the setup was cross-linked with glutaraldehyde (for 20 minutes). The excess of unbound protein was washed with phosphate (pH = 7.0), acetate (pH = 5.2) and Tris–HCl (pH = 7.2) buffers.

Enzyme immobilized by physical adsorption with a cross-linker does not require further activation. According to this procedure, a modified GCE/poly-4,4′-bBT/Tyr electrode was obtained (Fig. 2) and stored at 4 °C when not in use.

For the visualization of the topography of the GCE modified surface to determine the polymer film structure, as well as the bioplatform creation, atomic force microscopy (AFM) was adopted. All measurements were performed using Bruker MultiMode V microscope. The analyses were performed in tapping mode under air-ambient conditions (25 °C and 35% relative humidity) and with a scanning speed of 3 μm s^−1^. The standard etched rotated silicon tips were used with a tip radius <10 nm and nominal resonance frequency of 300 kHz cutting edge.

### 2.3. Electrochemical measurements

For EP determination, cyclic voltammetry (CV), differential pulse voltammetry (DPV), and chronoamperometry (CA) were applied with a potentiostat/galvanostat AUTOLAB PGSTAT128N with NOVA software. Measurements were carried out in a typical three-electrode system in a 10 mL cell. The GC electrode unmodified or modified with a thin polymer film of poly-4,4′-bBT and tyrosinase was used as a working electrode, a coiled platinum wire as an auxiliary electrode, and a silver–silver chloride electrode (Ag/AgCl) as a reference. CV measurements, showing the entire redox cycle, were carried out by repeated potential scanning in the range of −0.2–0.8 V. All electrochemical measurements were performed at a scan rate of 50 mV s^−1^, at room temperature under air-opened conditions. DPV measurements, used for linear range determination, were executed in the same potential range (−0.2–0.8 V). For comparison, the linearity was also calculated by CA at 0.3 V potential.

### 2.4. Electrochemical detection of epinephrine

The EP determination was conducted as described in Section 2.3. CV results were executed in a potential range from −0.2 to 0.8 V *vs.* Ag/AgCl for 3 cycles each, at a scan rate of 50 mV s^−1^. The DPV and CA analysis of EP were provided in the same potential range (−0.2–0.8 V) *vs.* Ag/AgCl. To analyze the possibility of the biosystem working under open-air conditions at room temperature, all electrochemical measurements were performed under such conditions. Substrate solutions of EP in a concentration range of 1–200 μM were prepared in 0.1 M PBS buffer at pH = 7.0 (the same conditions as during enzyme immobilization). For the measurements, the electrochemical cell was filled with 8 mL of fresh EP solutions. The current response was proportional to the given concentration.

### 2.5. Selectivity test

Interfering substances (ascorbic acid (AA), uric acid (UA), dopamine (DA), l-cysteine (CYS) and a mix of all interfering substances (MIX)) in the concentration of 50 μM were added to EP standard solution in the concentration of 25 μM (volume ratio 1 : 1) to test an influence of their presence (in excess in comparison to EP) on the analyte detection using DPV method.

### 2.6. Accuracy test

The EP detection accuracy test was performed using DPV analysis in a pharmaceutical drug – *Adrenalinum* WZF 300 μg/0.3 mL, produced by a pharmaceutical company Polfa Warszawa in Poland.

## 3. Results and discussion

### 3.1. Characterization of poly-4,4′-bis(2-methyl-3-butyn-2-ol)-2,2′-bithiophene and the bioplatform

Immobilization of an enzyme onto the electrode surface plays an extremely important role in the performance characteristics of biodevices, however, biological elements have to be directly attached to the surface of the transducer to obtain good sensitivity and a long operational life.^31^ Conductive polymers are widely used in the construction of biosensors due to their easy modification (which is fundamental for proper protein anchoring). Furthermore, they possess excellent electrical properties which allow bioinformation conversion into electrical response and therefore make it possible to obtain lightweight, ultra-conformable but also portable devices.^31,32^ What is more, conductive polymers act as mediators in electrochemical measurements by improving electron transfer between the enzyme's active center and the electrode. The electropolymerization of monomers to obtain redox-active and conductive polymer layers depends on the monomer's oxidation potential. In case of thiophene derivatives, high potential values during electrosynthesis may cause degradation of the polymer. To solve this inconvenience, thiophene units may be linked with aliphatic chains or hydroxylic groups, which reduce the oxidation potential to allow obtaining a stable polymer layer on the electrode surface.^33^

In this report, a polymer built of a thiophene derivative was electrochemically synthesized on the GCE surface in the presence of 0.1 M TBA-TFB supporting electrolyte.

The polymeric film based on a polythiophene derivative, obtained by electrodeposition, is bonded to the surface of the electrode *via* electrostatic interactions, due to mechanical interactions or decreasing the attack of the metal/polymer interface by electrolyte delamination.^34,35^ Such approach is suitable for building a conductive polymer that is strongly connected with the electrode surface. In addition, introducing some functional groups, such as –COOH, –OH, could improve electrical conductivity and, what is most important, allow for the protein immobilization onto its surface.

Polythiophene film was obtained using the cyclic voltammetry technique by cycling the potential in the range of 0–1.4 V and the polymerization lasted for 10 cycles. This number of cycles forms a stable polymer film with an appropriate thickness on the electrode surface. The electrosynthesis of poly-4,4′-bBT formed the electroactive polymer layer on the GCE surface. The optimal potential range for the oxidation potential of the monomer was experimentally determined with a DPV measurement in the potential range of 0–2 V *via* Ag/AgCl as a reference electrode. GC electrode modified with electrochemically synthesized polymer, as described above, was stable for 4 months and stored at 4 °C when not used, which was then confirmed with CV measurements. The temperature during measurements was room temperature, equal to 21 °C.

Onto the GC electrode, electrochemically modified with thin poly-4,4′-bBT film, the bioreceptor layer – tyrosinase – was immobilized according to the description in Section 2.2.

**Fig. 3:**
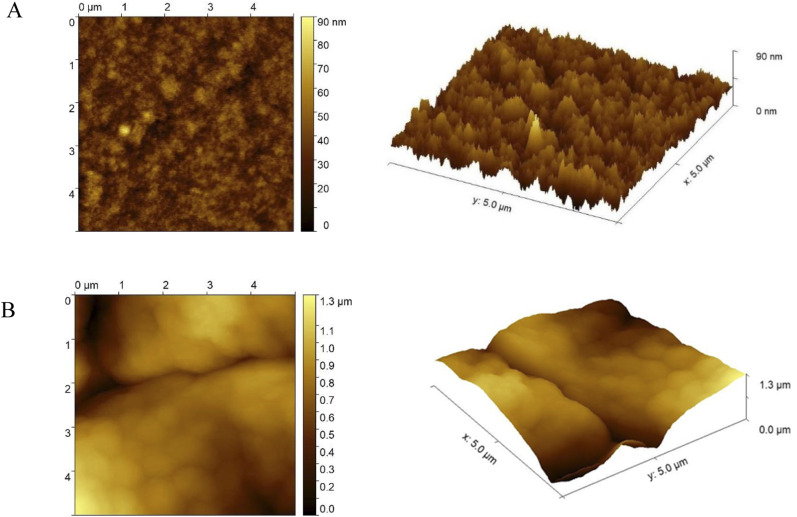
(A) Representative AFM topography maps and surface 3D views of electrode surfaces modified with poly-4,4′-bis(2-methyl-3-butyn-2-ol)-2,2′-bithiophene and (B) poly-4,4′-bis(2-methyl-3-butyn-2-ol)-2,2′-bithiophene with tyrosinase; 5 × 5 μm.

The morphology of modified electrodes with the thiophene derivative (GCE/poly-4,4′-bBT and GCE/poly-4,4′-bBT/Tyr) was analyzed using atomic force microscopy (AFM). The AFM topography maps and surface 3D views of the polymeric film and the biorecognition element are presented in Fig. 3.

Obtained polymer, visible in Fig. 3A, formed a highly globular, accurate, and very tightly packed layer with a relatively right structural integrity, as well as high stability of the film, indicating that protein can be successfully attached to the polymeric layer. To certify the effective immobilization of tyrosinase onto GCE modified with poly-4,4′-bBT, another AFM analysis was conducted. Results shown in Fig. 3B reveal that the enzyme covered the surface of the polymer and changed the morphology to a smoother, less globular, and less “strict” structure. The bioreceptor is responsible for the direct recognition of the analyte, therefore less rigid structure is very important, as it allows the enzyme to expose its active center and catalyze the specific reaction, which is the basis of the detection. Furthermore, as may be observed in Fig. 3, the thickness of the polymeric film is approximately 90 nm, but after protein anchoring, the thickness increases up to 1.3 μm.

### 3.2. Detection assay for epinephrine

**Fig. 4:**
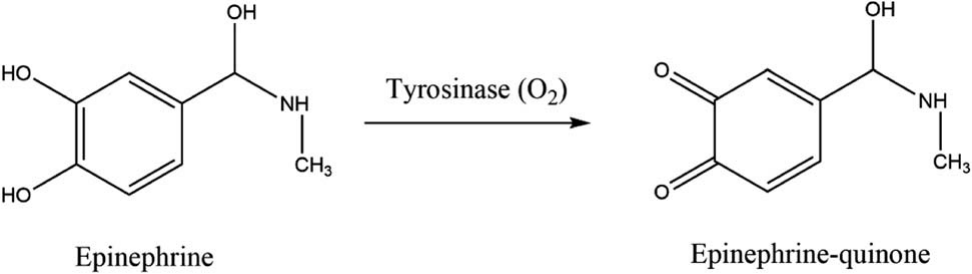
Redox reaction of EP catalyzed by tyrosinase.

Many analytical techniques for the determination of neurotransmitters are reported in the literature, for instance, high performance liquid chromatography (HPLC), capillary electrophoresis, spectrophotometry, or gas chromatography.^36–41^ Nonetheless, most of these procedures are expensive, do not allow continuous monitoring, are active only for a short time, or have low sensitivity and selectivity. They often require complex pre-treatment steps and expensive laboratory equipment. Electrochemical biosensors provide an inexpensive and easily operable analytical method for a selective, sensitive, and fast neurotransmitters detection with a potential for miniaturization.^42^ The electrochemical nature of epinephrine is pH-dependent, as was first observed by Ralph Adams in 1967.^43^ At physiological pH (near 7) and catalyzed by oxidoreductases, such as tyrosinase, epinephrine is oxidized to epinephrine quinone with the following loss of two electrons, as presented in Fig. 4, which can be electrochemically observed.

However, the electrochemical determination of neurotransmitters like epinephrine may be complicated. It is difficult to detect EP because of its rapid metabolism. Furthermore, at a bare GC electrode, it is oxidized at a potential near the most significant interferent species present in the human body, like ascorbic acid (AA) or uric acid (UA). The oxidized neurotransmitter can catalyze the oxidation of AA, which results in the generation of a single broad peak for both analytes.^42,44^ This is why selective determination of EP in the presence of typical interfering species is very often challenging. Another critical problem associated with the detection of catecholamines is the passivation of the electrode surface due to the polymerization of the oxidation products of catecholamines.^45,46^ A further crucial problem may be the passivation of the oxidation intermediates at the electrode surface during neurotransmitters measurements. To avoid mentioned inconveniences, a carbon electrode can be applied, like GCE with electrochemical pretreatment, due to its simplicity, efficiency, and low cost, as well as resulting in undistorted, well-defined, and reproducible signals.^47^ Another solution is based on using surface modifications that prevent passivation,^48^ such as modification of the electrode surface by fouling resistant, strongly absorbable polymers and by enzymes, which also improve the selectivity of biosensors (help avoid the influence of AA, UA).

In the research reported here, EP was determined in a wide concentration range (1–200 μM) using described detection system based on GCE/poly-4,4′-bBT/Tyr. EP solutions were prepared in PBS buffer (pH = 7.0) which is the most optimal for enzyme work.

**Fig. 5:**
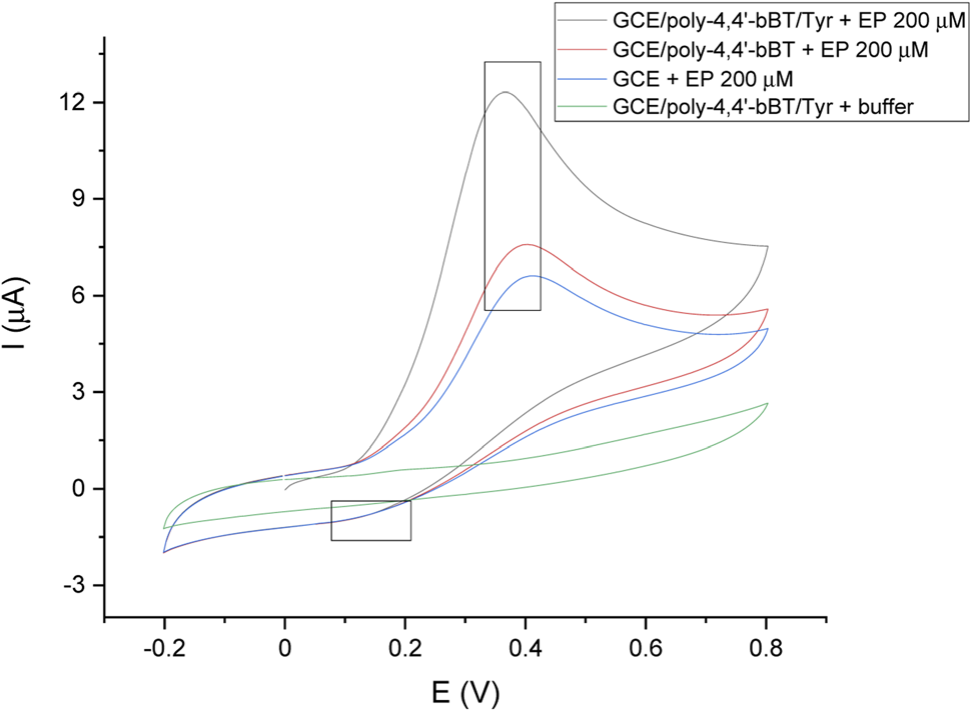
Representative CV scans of the bare GC electrode (blue line), GCE modified with poly-4,4′-bBT (red line), and GCE/poly-4,4′-bBT/Tyr (black line) in the presence of 200 μM EP, and GCE/poly-4,4′-bBT/Tyr (green line) in the absence of the analyte; applied potential range −0.2–0.8 V, scan rate 50 mV s^−1^, *vs.* Ag/AgCl.

Fig. 5 presents the voltammogram of a bare GC electrode, GCE modified with poly-4,4′-bBT, and the whole detection bioplatform GCE/poly-4,4′-bBT/Tyr, recorded in the presence of 200 μM EP using a cyclic voltammetry technique, which allows for observations of the whole redox process (potential range −0.2–0.8 V, scan rate 50 mV s^−1^). In addition, the background signal (from the buffer) is also presented in the voltammogram. Chosen potential range allows observation of EP oxidation reaction occurring in a potential range of 0.2–0.5 V (anodic direction). The signals of epinephrine oxidation at the bare GCE (blue line) are slightly visible, which indicates the lowest possibilities of EP redox reaction. The highest anodic peak (enzymatic oxidation of EP to EP-quinone) is observed for GCE modified with a polymer layer and enzyme presence (black line), at a potential value of 0.3 V, characteristic for EP at pH = 7.0, PBS buffer. It is generally known that the electro-oxidation of phenols is a barely reversible process with the subsequent blockage of the electrode by oxidation products, however a reduction of EP-quinone to EP is slightly visible as a cathodic small signal near 0.1 V, which may indicate a semi-reversible system, but in a very limited range. As can be observed, the constructed bioplatform GCE/poly-4,4′-bBT/Tyr showed the highest current value at 12 μA, which indicates high enzymatic activity for EP oxidation. Tyrosinase catalyzes the oxidation of epinephrine to epinephrine-quinone, which can then be electrochemically reduced at low potential values.^42^

To obtain operational parameters of biosensors, such as linearity and detection limit, differential pulse voltammetry (DPV) and chronoamperometry (CA) were used, and the results were compared. In DPV, the potential is scanned with a series of pulses and the current is measured at two points for each pulse, the first one just before the application of the pulse, and the second at the end of the pulse. These sampling points are selected to allow for the decay of the non-faradaic (charging) current, which results in higher sensitivity of this technique in comparison with CV (which is sensitive to residual current).^49^ In addition, many compounds, like phenols derivatives, are coupled to homogeneous chemical reactions, where the extent of charge transfer tends to increase for lower scan rates. This is why the relative contribution of phenols derivatives with slower coupled chemical reactions is higher for DPV than for CV.^50^ CA is a time-dependent technique, where a square-wave potential is applied to the working electrode.^51^ In this method, increases or decreases in the diffuse analyte layers at the working electrode surface cause changes in current values. By the IUPAC definition, the diffuse layer is equal to the surrounding region of an electrode in which the analyte concentrations are different from those in the bulk solution. When a proper potential is applied to the measurement system, the analyte's local concentration falls to zero. Under these conditions, the occurring gradient of the analyte's concentration supplies analyte diffusion from the bulk solution to the surface of the working electrode.^50,51^

**Fig. 6:**
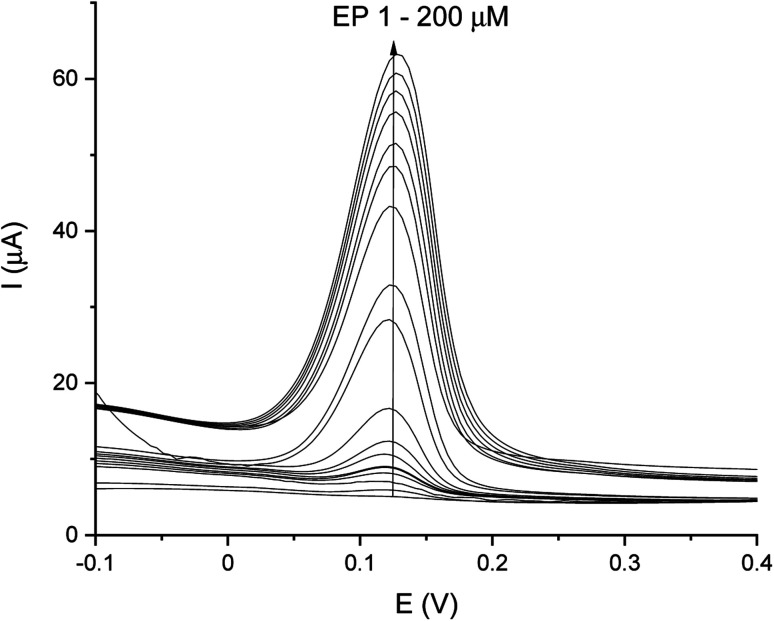
DPV-scans for different concentrations of EP in a range of 1–200 μM.

**Fig. 7:**
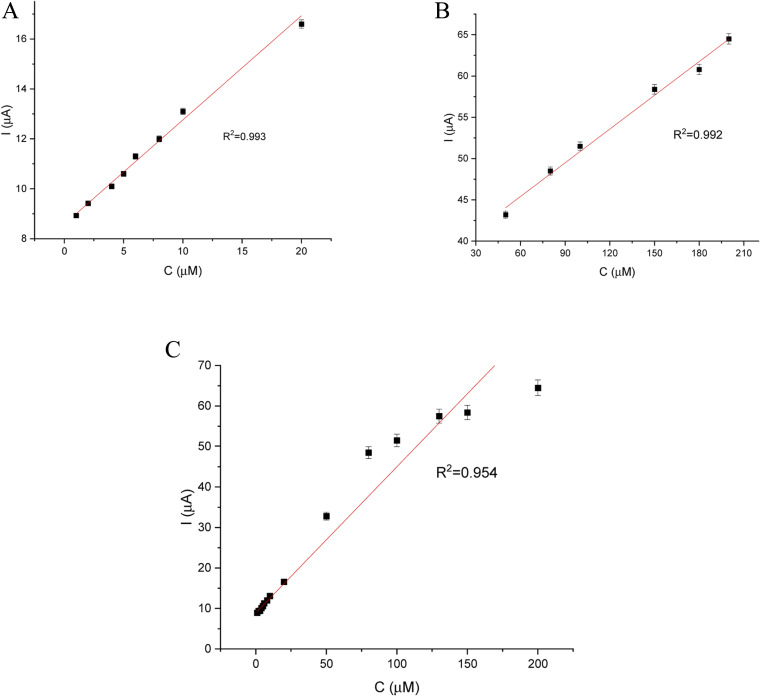
Relationship between EP concentration and current (biosensor response): (A) – in a low concentration range (1–20 μM), (B) – in a high concentration range (30–200 μM), (C) – in a full concentration range (1–200 μM).

DPV and CA results were compared and validation parameters were obtained. A DPV voltammogram is presented in Fig. 6, where oxidation signals precisely correspond to the given EP concentration. Changes in current increase proportionally with the concentration of EP in a range of 1–200 μM. Fig. 7A represents the linearity based on the DPV method of the GCE/poly-4,4′-bBT/Tyr for a lower concentration range (1–20 μM) with an excellent linear response to EP (linear coefficient *R*^2^ = 0.993). In addition, Fig. 7B shows the linearity obtained for a higher concentration range (30–200 μM), where a linear response of EP is observed with a linear coefficient almost as high as in the lower concentration range (*R*^2^ = 0.992). Both results gave a highly linear response (*R*^2^ close to 1) to EP and show that the present analytical biosystem is suitable in a wide concentration range. Further parameters for the analytical validation are shown in Table 1.

**Table 1:** Analytical parameters of the calibration straight of GCE/poly-4,4′-bBT/Tyr with the DPV technique

Linear range	*R* ^2^	Slope (*a*)	SD of slope	Intercept (*b*)	SD of intercept
1–20 μM	0.993	0.408	1.37 × 10^−3^	8.651	0.1
30–200 μM	0.992	0.136	0.67 × 10^−3^	37.24	0.8

**Fig. 8:**
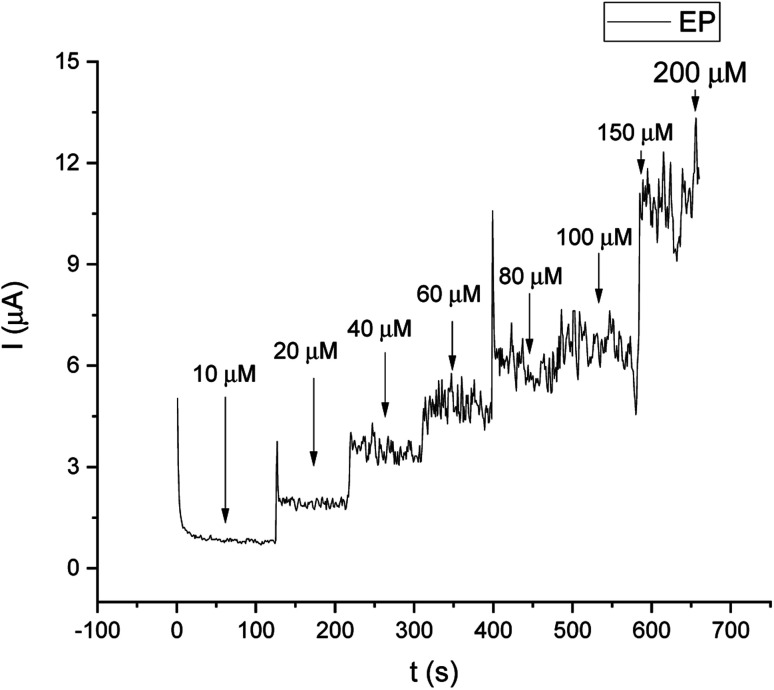
Current/time response of different concentrations of EP in the range of 10–200 μM.

**Fig. 9:**
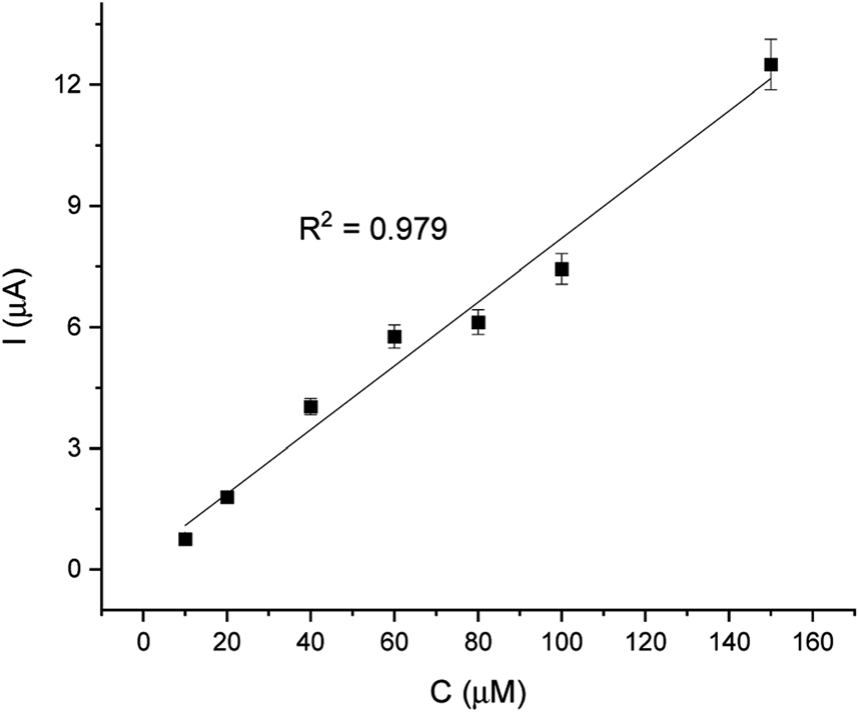
Linear relationship of current and EP concentration (10–200 μM).

For comparison, the CA technique was employed for testing the linear response of EP in a range of 1–200 μM. The current/time response of the biosensor GCE/poly-4,4′-bBT/Tyr is presented in Fig. 8. First of all, high noise is seen during the measurements, increasing with the EP concentration. Furthermore, low concentrations of EP (1–9 μM) were not detectable. Obtained results may be caused by the fact that sampled current value is dependent on the sampling time. The application of CA may be problematic for distinguishing different concentrations by two overlapping *I*–*t* curves at the steady state.^52^ As happened in this case, in CA the sample value may be out of the measurement range. Besides the fact that CA allows for time-dependent monitoring, in order to obtain high signal/noise values (mainly at low concentration values) and a wide linear range, the DPV method showed much better reproducibility, making the DPV experiment reliable. This is why the DPV method was chosen for the investigation of the behavior of the biosensor. Fig. 9 shows the dependence of current on EP concentration measured by CA obtained in a range of 10–200 μM, with *R*^2^ = 0.979. As can be observed, in the concentration range of 10–60 μM, the current signal is proportionally increasing with the concentration of EP, suggesting a first order reaction. At further increases in epinephrine concentration (60–120 μM), the current increases slowly, and the enzymatic reaction may show a transition to a zero-order reaction,^53^ which confirms results obtained from the DPV technique for this biosystem based on tyrosinase.

Analytical parameters of obtained slope from the CA method are presented in Table 2.

**Table 2:** Analytical parameters of the calibration straight of GCE/poly-4,4′-bBT/Tyr with the CA technique

Linearity	*R* ^2^	Slope	SD of slope	Intercept	SD of intercept
10–200 μM	0.979	0.179	1.16 × 10^−3^	3.012	0.4

Moreover, from the calibration data, the Hill coefficient (*h*) can be calculated to check the enzymatic activity of the designed system GCE/poly-4,4′-bBT/Tyr. This parameter reflects the binding of analytes by the enzyme's active center; in other words, it may inform about the cooperative effect between the occupied active sites.^54^ The Hill coefficient was calculated by representing log[*I*/(*I*_max_ − *I*)] *vs.* log[C] (logarithm of the analyte concentration) of the enzymatic oxidation of EP on the electrode surface, from the DPV method (in higher ranges). Obtained result is very close to unity, indicating a positive cooperative effect (*h* = 1.04 ± 0.02; *R*^2^ = 0.989), and allows calculating other kinetic parameters from the Michaelis–Menten kinetics, where enzyme-based reaction fits. Adapted Lineweaver–Burk eqn (1) was used for the calculation of the Michaelis–Menten constant and the maximum current response:1
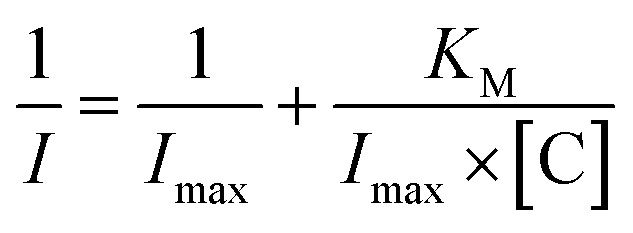
where *I* is the current, *I*_max_ is the steady-state current, *K*_M_ is the apparent Michaelis–Menten constant, and [C] is the concentration of the analyte (EP).^49^*I*_max_ and *K*_M_ values were estimated from the intercept (*I*_max_) and slope (*K*_M_) and were equal to 0.27 μA and 53.18 μM, respectively. The small value of *I*_max_, which is the maximum current under saturated substrate conditions (corresponds to the maximum rate of enzymatic reaction *V*_max_, according to Michaelis–Menten kinetics), and the high value of *K*_M_, indicate high sensitivity of constructed biosensor system.^49^

The detection limit was calculated as:2
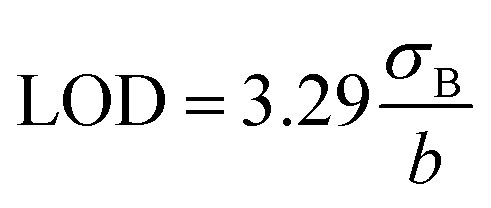
where *σ*_B_ is the standard deviation of the population of blank responses and *b* is the slope of the regression line.^55^ For the low concentration range (1–20 μM), the theoretical, calculated LOD was equal to 0.18 nM, whereas for the high concentration range (30–200 μM), it was equal to 1.03 nM for the DPV analysis and 125 nM in case of CA. However, the LOD obtained based on the results is equal to the minimum concentration of the analyte – 1 μM – that was determined with the constructed bioanalytical system. Obtained detection limits are very promising in case of real EP determination. The physiological level of EP in body fluids is 0.7 nM, however, in the presence of cardiovascular disorders, the concentration may increase up to 500 nM.^56,57^ EP monitoring is extremely important for diabetes and patients with cardiological disorders due to its function as a regulator of blood pressure, glucose, and water/fluid imbalance.^58,59^ Hence, obtained values of LOD are very promising in this context and are better than other biosensor systems described in the literature (Table 3).

**Table 3:** Comparison of biosensors for EP detection; GCE – glassy carbon electrode, Tyr – tyrosinase

	Biosensor/sensor	Technique	Linear range	LOD	Ref.
1	GCE/CuO nanorods	DPV	0.04–14 μM	20 nM	60
2	Au/poly-4,7-bis(5-(pyridin-2-yl)thiophen-2-yl) benzo[*c*][1,2,5]thiadiazole/Tyr	CA	0.1–50 μM	0.06 μM	61
3	CP/MWCNT/Tyr/Nafion	DPV	5 μM–50 mM	300 nM	62
4	GR/Au/GCE	CV	50 nM–8 μM	7 nM	63
5	GCE/poly-4,4′-bBT/Tyr	DPV	30–200 μM	1.03 nM	This work

The limits of quantification (LOQ) were also determined (calculated using eqn (3)) and were equal to 0.49 nM (DPV, low concentration range), 2.77 nM (DPV, high concentration range), and 337.5 nM (CA technique).3
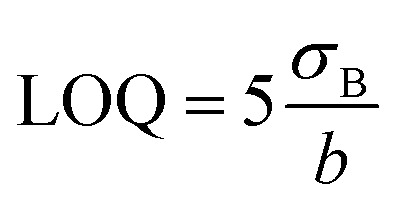
where *σ*_B_ is the standard deviation of the population of blank responses and *b* is the slope of the regression line.^55^ Furthermore, the sensitivity of the proposed biosensor, calculated as the slope of the linearity graph divided by the geometry/active area of the biosensor, was found to be 0.0011 μA mM^−1^ cm^−2^. Another parameter was calculated for total enzyme surface coverage, using the Laviron eqn (4):4
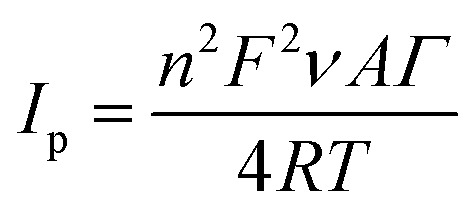
where *I*_p_ is the peak current (A), *n* is the number of electrons, *F* is the Faraday constant (96 485.3365 C mol^−1^), *ν* is the potential scan rate (V s^−1^), *A* is the electrode area (cm^2^), *Γ* is the surface coverage (mol cm^−2^), *R* is the ideal gas constant (8.3144621 J K^−1^ mol^−1^), *T* is the temperature (K).^49^

Obtained value of the total surface coverage was 4.18 × 10^−12^ mol cm^−2^, which stays in agreement with the literature data.^53^ In addition, immobilization of the enzyme onto the polymer surface preserves a high catalytic activity of tyrosinase.

To examine storage stability, DPV analysis of GCE/poly-4,4′-bBT/Tyr in 200 μM EP solution under optimal conditions (0.1 M PBS at pH = 7.0) was performed. The biosensor was stored at 4 °C when not in use. The measurements were recorded over 60 days. On the first day, the current response of the biosensor was accepted as 100%, and after 45 days the biosensor activity remained at 73%. After 60 days the current response was 57% of the initial value. The stability of described biosensor was 49 days, when the signal obtained a value of 70% of the initial response.

**Fig. 10:**
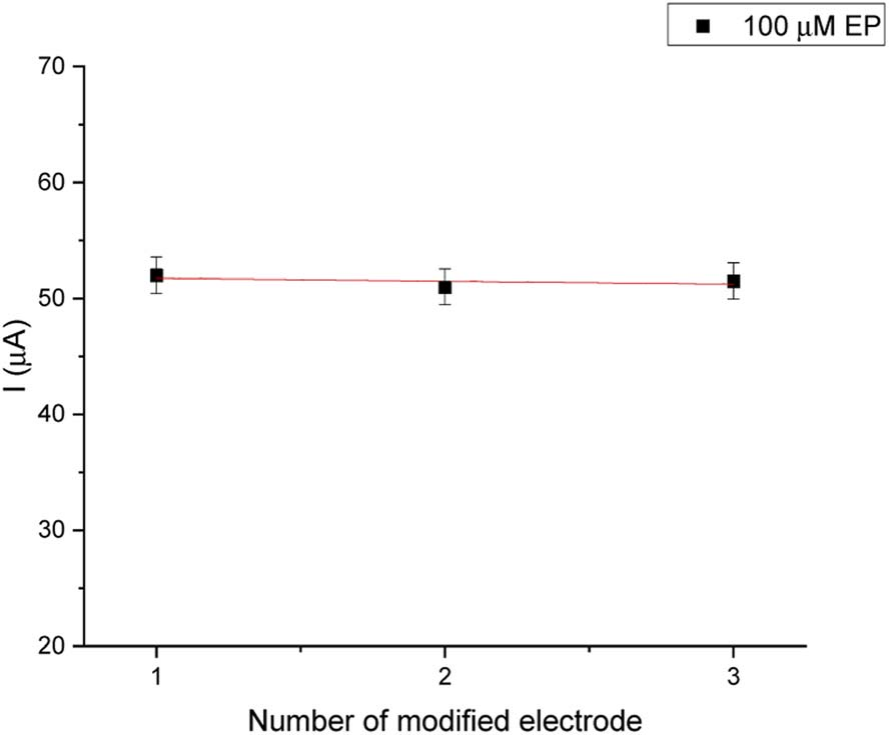
Reproducibility of the biosensor.

The reproducibility of electrode-to-electrode was tested by preparing three biosensing systems under the same conditions (Fig. 10). These experiments were conducted under optimal experimental conditions for 100 μM EP (0.1 M PBS, pH = 7.0). The relative standard deviation (RSD%) was obtained as 2.7%.

### 3.3. Selectivity test

**Fig. 11:**
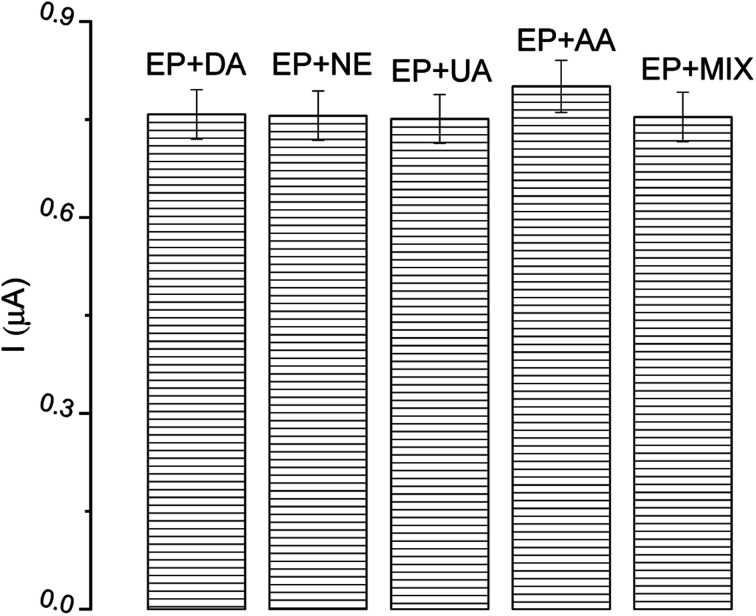
The effect of interfering substances (in excess) on epinephrine (EP) determination; DA – dopamine, NE – norepinephrine, UA – uric acid, AA – ascorbic acid.

In the construction of biosensor devices, selectivity is a vital operational parameter. It provides information on the possibility of the detection of only the interesting analyte from a mixture. The most common compounds present in human body fluids, as well as species which possess similar oxidation potential and similar structure to EP, were investigated as possible interfering substances, *i.e.* dopamine (DA), norepinephrine (NE), uric acid (UA), ascorbic acid (AA) and a mix of all analyzed species. Solutions of each compound were added (in twice the excess) to solutions of EP. The current response from the DPV technique was compared to the signal obtained from EP. As can be seen in Fig. 11, there is a negligible effect of the interfering species on the EP determination, confirming the high selectivity of described tyrosinase-based biosensor. All tested reagents had a negligible effect (<1.3%) on the peak current of the samples compared to the blank (0.7% DA, 1.1% NE, 0.6% UA, 1.6% AA, 1.2% MIX).

### 3.4. Accuracy test

The real sample analysis was conducted with a pharmaceutical product – a drug *Adrenalinum* WZF produced by Polfa Warszawa, Poland. The accuracy of proposed method gives information about the practicability of the introduced study for future application. The drug was diluted to obtain the final concentration of 50 μM in 0.1 M PBS buffer (pH = 7.0). Recovery and RSD values are reported in Table 4. Obtained recovery value (calculated as a ratio of detected concentration to the real concentration of EP in the real samples (%)) is very good (98%) and demonstrates that the proposed strategy is suitable for the real detection of this neurotransmitter. Biosensors need particular and careful calibration to reach satisfactory accuracy during fabrication.^61^ The Clinical and Laboratory Standards Institute (CLSI; EP05-A3, EP24-A2, EP25-A) demands a value of the variation coefficient less than 10% for parameters such as accuracy, stability, and reproducibility.^62^ In case of our study, obtained accuracy value fits these standards. For reaching a high accuracy value in biosensors, crucial steps are proper modification regarding the electrode and the use of a proper mediator – presented here enzymatic biosensor offers easy to prepare and direct interaction (without the need of a label) with the investigated analyte, which increases the accuracy of described bioanalytical system.^63^

**Table 4:** Results obtained for EP determination based on proposed method

Concentration of EP in a real sample (μM)	*C*_detected_ (μM)	Recovery (%)	RSD (%)
50.00	49.12	98	±1.32

## 4. Conclusions

The crucial problem in the design of enzymatic electrodes is to enhance the speed and reversibility of charge transfer between the enzyme and the electrode. Organic polymers are the most versatile and effective molecular scaffolds for functional materials with significantly improved optical and electronic properties. In this work, a polymer based on a thiophene derivative, poly-4,4′-bis(2-methyl-3-butyn-2-ol)-2,2′-bithiophene (poly-4,4′-bBT), on a GC electrode was used as a semi-conductive, electron-mediated matrix for tyrosinase anchoring. Tyrosinase was used as a biorecognition element for selective and sensitive determination of epinephrine. The biodetection platform for EP determination was then constructed as GCE/poly-4,4′-bBT/Tyr, which was confirmed by AFM analysis. EP was detected using DPV and CA techniques, which demonstrate very good operational parameters of described biosensing assay. The DPV technique presented a wide linear range (1–20 μM and 30–200 μM) and a low detection limit (0.18 nM and 1.03 nM for the lower and higher concentrations, respectively). In case of chronoamperometry, a high signal-to-noise ratio and lower reproducibility were observed, causing a less broad linear range (10–200 μM) and a higher detection limit (125 nM). Therefore, a DPV technique was used for the calculation of sensitivity (0.0011 μA mM^−1^ cm^−2^), stability (49 days), and total surface coverage (4.18 × 10^−12^ mol^−1^ cm^2^). The biosensor also showed a very high selectivity in the presence of common interfering species (*i.e.* AA, DA, NE, UA), as all examined interfering substances have a slight effect on the signal during amperometric measurements (≤1.3%). Obtained biosensor was successfully validated for the proposed strategy of a pharmaceutical formulation (recovery value of 98.24%). All these characteristics establish a convenient, stable, simple, and long-term technique for epinephrine detection that could be recommended as an excellent bio-tool for diagnostic purposes.

## Conflicts of interest

There are no conflicts to declare.

## Supplementary Material
